# A Case of Schizophrenia in a Young Male Adult with no History of Substance Abuse: Impact of Clinical Pharmacists' Interventions on Patient Outcome

**DOI:** 10.1155/2020/3419609

**Published:** 2020-02-15

**Authors:** Mercy N. A. Opare-Addo, Josephine Mensah, Grace Owusu Aboagye

**Affiliations:** ^1^Department of Pharmacy Practice, Kwame Nkrumah University of Science and Technology (KNUST), PMB, U.P.O., Kumasi, Ghana; ^2^Department of Surgery, Pharmacy Unit, Korle Bu Teaching Hospital, P.O. Box 77, Accra, Ghana; ^3^Department of Psychiatry, Korle Bu Teaching Hospital, P.O. Box 77, Accra, Ghana

## Abstract

Schizophrenia is a chronic and severe mental disorder characterized by distortions in thinking, perception, emotions, language, sense of self, and behaviour. This report presents the role of clinical pharmacists in the management of a patient diagnosed with schizophrenia with symptoms of paranoia. A gainfully employed young African male adult reported to be roaming around town moving from one bank to another was arrested. The patient was referred to the psychiatric unit of a hospital and diagnosed with schizophrenia. Key interventions offered included rapid tranquilization, electroconvulsive therapy, and psychotherapy. Medications administered to the patient while on admission included IV diazepam, IM haloperidol, IV Ketamine, IM flupentixol, olanzapine tablets, and trihexyphenidyl tablets. Issues raised by clinical pharmacists during the patient's admission included need for alternative medication for rapid tranquilization, need for initial investigations and documentation of the patient's vitals, initiation of antipsychotic therapy without initial monitoring and screening for substance abuse, inappropriate dose at initiation of antipsychotic medications, untreated indication, and incidence of missed doses. Interventions by the clinical pharmacists contributed to improvement in the patient's symptoms prior to hospital discharge. The case proves that it is critical for clinical pharmacists to be involved in the multidisciplinary team during management of patients with psychosis.

## 1. Introduction

Schizophrenia is a chronic and severe mental disorder affecting more than 21 million people worldwide which is characterized by distortions in thinking, perception, emotions, language, sense of self, and behaviour. Common experiences include hallucinations mostly involving hearing voices or seeing things that are not there and delusions which involve having fixed, false beliefs [1].

Since schizophrenia is a chronic illness that influences virtually all aspects of life of affected persons, treatment planning has three goals which are to reduce or eliminate symptoms, to maximize quality of life and adaptive functioning, and to promote and maintain recovery from the debilitating effects of illness to the maximum extent possible [2, 3].

Medications are invaluable in the management of patients with mental illnesses. Pharmacists are therefore indispensable in improving the quality of service rendered to patients with mental illnesses such as schizophrenia which contributes to reduction of the numerous problems associated with and faced by patients with mental disorders [4].

Management of patients with conditions such as schizophrenia is generally a collaborative effort which encompasses incorporation of skills of a myriad of health care professionals involved in patient care. Clinical pharmacists have been instrumental in several roles such as being educators, consultants, and providers for over 30 years [5]. Since pharmacists are authorities in pharmaceutical care, they also apply their complementary skills and knowledge in managing patients with mental illnesses together with other health care professionals in the multidisciplinary team [4]. Clinical pharmacists as such contribute to patient care by playing a vital role in the detection, resolution, and prevention of medication-related problems. In ensuring the safe and efficacious use of medications, clinical pharmacists are also pivotal. In addition, pharmacists are available to provide comprehensive drug information to patients with mental illnesses such as schizophrenia, the patient relatives, and other health care professionals involved in patient management. Pharmacists spearhead medication adherence and are involved in education on primary prevention of mental illnesses, health promotion, and lifestyle modification [4].

In a review of clinical pharmacy services offered in mental health care conducted by Richardson et al., 18 hospital-based studies were identified in which interventions conducted by clinical pharmacists during patient medication chart reviews, laboratory investigation result assessment, and prescription of medications were identified. In these studies, clinical pharmacists also educated the patients with mental disorders and other health care professionals [6].

Another systematic review which evaluated the quantity and quality of medical literature examining the impact of pharmacists in mental health care from 1972 to 2003 reported that there were improvements in outcomes, prescribing practices, patient satisfaction, and resource use when pharmacists are involved in mental health care [5].

We report a case involving a young African male adult with gainful employment and no history of substance abuse who was diagnosed with schizophrenia. The patient was referred to the psychiatric unit of a hospital, and interventions by clinical pharmacists during management of the patient's medical problems contributed to overall improved outcomes before hospital discharge.

## 2. The Case

An adult male who was seen at a psychiatric unit complained of hearing voices for the past 10 months and loss of appetite. He also complained that he roamed around town, and even though he returned home, he was sometimes physically abusive to his mother and sister. The patient further added that he felt unsafe and thought that people wanted to harm him.

The patient explained that he took a leave from work because he was feeling feverish and asked someone to replace him, but he never returned to the workplace. A month after commencing leave, he received his salary for work done but subsequently was not paid by his company. He decided to move from one bank branch to another, trying to make withdrawals. On his third attempt at one branch, he was arrested and put in cells for four days, but he was never given any reason. He said he had also received death threats, one of which was a call from an unfamiliar number. No words were spoken, but he perceived that it was a signal that meant that his life was to be taken.

His social history revealed that he lived in the city with his mother and sister. He had a girlfriend who stayed at a different suburb of the city. His past medical history included a history of paranoid delusions. There was no family history of mental or physical illness. He had a premorbid personality of being introvert.

When the patient was first seen at the psychiatric unit, relevant signs included complaints of difficulty in sleeping, loss of appetite, roaming around town, and sometimes being physically abusive to his mother and sister for the past 10 months. Mental state examination conducted upon hospital admission revealed that the patient was emaciated and appeared informally dressed. He exhibited apprehensive behaviour, fatuous laughter, and hesitancy. He also paced to and fro on the ward. His mood was depressed and affect constricted. The patient experienced auditory hallucinations. He complained of hearing voices and engaged in third person conversations involving both a man and a woman. The patient also complained that he heard people talking about him and insulting him, some of whom he knew. He was therefore suspicious and felt uneasy with others. As a result, the patient said that he had been spending the night with a friend and refused to return to his parents' house because he was not safe there. He believed that he had been targeted. His thought content revealed paranoid delusions and delusions of reference. (The patient's sister said he often complained that people wanted to harm him and as such felt unsafe. She also added that he often complained of receiving death threats through phone calls from an unfamiliar telephone number.) The patient also had poor insight.

His risk factors included being born and raised in an urban area and male sex which put him at risk of paranoid schizophrenia. When seen at the psychiatric unit, he underwent electroconvulsive therapy which also put him at risk of experiencing tonic-clonic seizures.

The patient had not taken prescription medications in the past six months prior to being seen at the hospital since he had not been diagnosed with any mental illness previously. He had no known allergies, and his lifestyle information revealed that he did not smoke or use recreational drugs. The patient demonstrated suspected adherence concerns since he had poor insight and repeated several times that there was nothing wrong with him. His medications were therefore administered under directly observed therapy (DOT) while he was on admission.

The patient's blood samples showed that his renal function test, lipid profile test, liver function test, and full blood count test conducted on the third day of hospital admission were within normal limits. The patient underwent psychotherapy (insight-oriented therapy) on the third day of hospital admission, and the report indicated that he was preoccupied with spirituality and believed his symptoms were spiritual. He said that the reason why he went to the hospital was between him and his girlfriend who sent him for admission. A second insight-oriented therapy conducted a week after hospital admission revealed that he still believed that he had no problem and desired to be discharged. The patient's medical problems were therefore identified as schizophrenia—a first episode psychosis in which he exhibited signs and symptoms of paranoia, insomnia, and anorexia.

Initially, the patient was not screened for substance abuse. The clinical pharmacists recommended on the third day of hospital admission that he should be screened for substance abuse to ascertain whether he had not engaged in any illicit drug use prior to the onset of his current symptoms.

The patient was initially administered a dose each of IM haloperidol 10 mg and IV diazepam 10 mg for rapid tranquilization when he was admitted due to aggression. The combination of IM haloperidol 10 mg and IM diazepam 10 mg was administered again after 24 hours, but both did not yield favorable results. His medication was subsequently changed to IM midazolam 7.5 mg and IM haloperidol 5 mg after the intervention of the clinical pharmacists. This combination was administered on the third day of admission, and the patient's aggression subsided. On the day of admission, the patient was also prescribed olanzapine 10 mg tablets for management of schizophrenia. However, on the second day of hospital admission, the patient complained of experiencing fine tremors due to initiation of olanzapine. The patient however had not been prescribed any medication for management of the fine tremors. This was considered as an untreated indication. He was therefore administered trihexyphenidyl 5 mg tablet upon the recommendation of the clinical pharmacists. The dose of olanzapine was also reduced to 5 mg upon recommendation by the clinical pharmacists.

The patient underwent four cycles of electroconvulsive therapy with IV Ketamine 500 mg and IV haloperidol 10 mg. These were done on the 2^nd^, 13^th^, 16^th^, and 18^th^ days of hospital admission. The patient's mood, appearance, behaviour, perception, and insight improved while on therapy. Although he was occasionally restless, he was mostly cooperative.

Although the patient's medications were administered under DOT, nurses complained that the patient refused his olanzapine 5 mg tablet on few days he was restless and uncooperative which resulted in incidence of missed doses. Nurses were encouraged by the clinical pharmacists to ensure continuous administration of tablet olanzapine 5 mg to the patient as prescribed to prevent relapse.

He was scheduled to be discharged on IM flupentixol 40 mg every 4 weeks and olanzapine 5 mg tablet daily. The clinical pharmacists intervened for the dose of IM flupentixol to be reduced to 20 mg. Therefore, the patient was discharged after 25 days of hospital admission on IM flupentixol 20 mg every 4 weeks and olanzapine 5 mg tablet daily (to be tapered off in 3 months). The patient however failed to report to the hospital for a psychiatric follow-up. As such, we were unable to obtain specific written consent from him for the case report. We have therefore omitted specific details about his age and occupation in order to preserve anonymity.

## 3. Discussion

This case report presents the role of clinical pharmacists in the management of a patient diagnosed with schizophrenia who demonstrated symptoms of paranoia. Issues raised by the pharmacists during the patient's admission included need for alternative medication for rapid tranquilization, need for initial investigations and documentation of the patient's vitals, initiation of antipsychotic therapy without initial monitoring and screening for substance abuse, inappropriate dose at initiation of antipsychotic medications, untreated indication, and incidence of missed doses. A study conducted on the implementation of clinical pharmacy services in a tertiary psychiatric hospital in Saudi Arabia also revealed similar findings, where care issues identified included duplication of drug therapy, drug interactions, inappropriate medication selection, dosing problems, and need for therapeutic drug monitoring [7]. In a prospective analysis of clinical pharmacy interventions on an acute psychiatric inpatient unit, a total of 204 clinical interventions were identified in 69 patients some of which included a recommendation for commencement of medication therapy and need for enhancement of patient monitoring [8]. A review of clinical pharmacy services offered in mental health also revealed that interventions were made by clinical pharmacists during patient medication chart reviews, laboratory investigation result assessment, and prescription of medications [6].

Recommendation for initial investigations and documentation of the patient's vitals on admission was consistent with recommendations by the practice guidelines for the treatment of patients with schizophrenia and National Institute for Health and Clinical Excellence (NICE) guidelines for prevention and management of psychosis and schizophrenia in adults. The practice guidelines for the treatment of patients with schizophrenia and NICE guidelines for prevention and management of psychosis and schizophrenia in adults also recommend that other causes of psychosis should be ruled out initially with electrocardiogram and by screening the patient for substance abuse while on admission [2, 9, 10].

According to the NICE guidelines for prevention and management of psychosis and schizophrenia in adults and the Indian Psychiatric Society treatment guidelines for the management of schizophrenia, acute behavioural disturbances in patients with schizophrenia such as aggression, agitation, or violence towards others due to delusions, hallucinations, and anxiety require urgent treatment. Therefore, rapid tranquilization is recommended for such patients [9, 11]. The recommendation by the clinical pharmacists, however, that the choice of IM haloperidol 10 mg and IV diazepam 10 mg was not optimal was consistent with guidelines for intramuscular medication for acute behavioural disturbance in mental health and learning disability and the clinical practice guidelines for the management of schizophrenia [11, 12]. A review of current data concerning management of agitated or aggressive patients as well as an open-label randomized controlled trial conducted by Huang et al. support the use of lorazepam plus haloperidol in acute psychotic agitation [13, 14]. Further administration of a second dose of IM haloperidol 10 mg and IM diazepam 10 mg when the initial administration of the same combination of medications did not offer favorable response was not in conformity with NICE guidelines for prevention and management of psychosis and schizophrenia in adults and the Indian Psychiatric Society treatment guidelines for the management of schizophrenia [9, 11].

The clinical pharmacist intervention that initiation of olanzapine tablets and IM haloperidol occurred without initial monitoring of patient parameters such as the patient's weight plotted on a chart, waist circumference, pulse and blood pressure, fasting blood glucose, glycosylated haemoglobin (HbA1c), blood lipid profile, liver function test, renal function test, and prolactin levels was in conformity with the APA and the Scottish Intercollegiate Guidelines Network (SIGN) guidelines for management of schizophrenia. According to these guidelines, since antipsychotic medications are associated with a range of adverse effects which can influence physical health, monitoring of patients at the baseline and subsequently based on a systematic evaluation of guidelines is of a great necessity [2, 15]. According to Muench and Hamer, all antipsychotic medications are associated with an increased likelihood of sedation, sexual dysfunction, postural hypotension, cardiac arrhythmia, and sudden cardiac death, and thus, patients require enhanced monitoring [16]. A prospective study conducted also revealed that recommendation for enhancement of patient monitoring was among the most common type of intervention made by clinical pharmacists in an acute psychiatric inpatient unit [8].

According to the British Association of Psychopharmacology, there is evidence that first episode psychosis responds to lower doses of antipsychotic medications. In addition, there is a biological sensitivity to antipsychotic medications in the early stages of the illness which applies to both the therapeutic and adverse effects [10]. The Joint Formulary Committee also recommends in the British National Formulary (BNF) that in the absence of a prior renal function test and liver function test, a dose of olanzapine 5 mg tablet should be administered, and IM flupentixol should be initiated at a dose of 20 mg [17]. The recommendation for reduction of the dose at which tablet olanzapine and IM flupentixol were initiated from 10 mg and 40 mg to 5 mg and 20 mg, respectively, was in conformity with these guidelines. This was also consistent with recommendations by Stroup and Marder that first episode schizophrenic patients require a lower medication dose with consideration of a gradual decrease or discontinuation at 6 months to 1 year [18].

The patient complained of fine tremors upon initiation of olanzapine therapy which was documented in his medical records. However, the patient had not been prescribed any medication for management of the fine tremors. This was considered as an untreated indication. The clinical pharmacists' intervention for the use of a separate formal side effect checklist for documentation of adverse effects associated with antipsychotic medication use was consistent with the British Association for Psychopharmacology's evidence-based guidelines for the pharmacological treatment of schizophrenia [10]. Intervention for the addition of trihexyphenidyl 5 mg tablet to the patient's medications was in agreement with Lerner et al. and the Joint Formulary Committee which recommend the use of antimuscarinic medications such as trihexyphenidyl 5 mg tablet for the management of acute extrapyramidal side effects associated with antipsychotic medication use [16, 19]. Intervention by the clinical pharmacists for continuous administration of tablet olanzapine to the patient as prescribed to prevent incidence of missed doses was consistent with guidelines for management of schizophrenia and several other studies conducted on antipsychotic medication use which recommend continued administration of antipsychotic medications in order to prevent relapse [9, 20, 21].

As a result of the collaboration between the clinical pharmacists and the multidisciplinary team in management of the patient, his mood, appearance, behaviour, perception, and insight improved eventually. He was therefore discharged after 25 days of hospital admission. The case highlights the fact that it is critical for clinical pharmacists to be involved in the multidisciplinary team during management of patients with psychosis since interventions by clinical pharmacists contribute to the overall improved patient outcomes.

## Abbreviations

APA: American Psychiatric Association
DOT: Directly observed therapy
DSM 5: Diagnostic and Statistical Manual of Mental Disorders, Fifth Edition
ECT: Electroconvulsive therapy
EUFEST: European First Episode Schizophrenia Trial
HbAlc: Glycosylated haemoglobin
ICD-10: International statistical classification of diseases and related health problems
IM: Intramuscular
IV: Intravenous
NICE: National Institute for Health and Clinical Excellence
SIGN: Scottish Intercollegiate Guidelines Network.
